# AI‐Powered Smartphone Application for Measuring Hallux Valgus Angle From Radiographs Displayed on a Monitor

**DOI:** 10.1002/jfa2.70081

**Published:** 2025-09-04

**Authors:** Ryutaro Takeda, Sanehiro Ando, Toshiko Iidaka, Kenta Makabe, Taro Kasai, Yasunori Omata, Noriko Yoshimura, Sakae Tanaka, Takumi Matsumoto

**Affiliations:** ^1^ Department of Orthopaedic Surgery Faculty of Medicine The University of Tokyo Tokyo Japan; ^2^ Department of Preventive Medicine for Locomotive Organ Disorders 22nd Century Medical and Research Center Faculty of Medicine The University of Tokyo Tokyo Japan

**Keywords:** artificial intelligence, automatic measurement, hallux valgus angle, intermetatarsal angle, neural network, smartphone

## Abstract

**Introduction:**

We developed a smartphone application capable of automatically measuring the hallux valgus angle (HVA) and various intermetatarsal angles by capturing radiographic images displayed on a monitor. This study aimed to evaluate the accuracy of these measurements using the application.

**Methods:**

Three users—a board‐certified orthopedic surgeon, a resident, and a nonhealthcare professional (Users 1, 2, and 3)—independently used the application to measure angles on 92 radiographs from 92 consecutive patients. Mean absolute errors (MAEs) between the application‐based measurements and the median of manual measurements performed by three experienced foot and ankle surgeons using a DICOM viewer were calculated for each user. To evaluate whether the measurement errors were acceptably small, one‐sided t‐tests were conducted to determine whether the MAEs were significantly less than 3°. Differences in MAEs among the three users were also assessed using analysis of variance.

**Results:**

The MAEs of HVA by the three users were 1.1°, 1.3°, and 1.4°, respectively, all significantly below the 3° threshold (95% CI upper limit; 1.2°, 1.5°, and 1.5°). Comparable accuracy was observed for intermetatarsal angles, which have slightly greater variability for more lateral metatarsals. All measurements met the accuracy criterion of < 3°, except for the intermetatarsal angles between the first and fifth metatarsals measured by the nonhealthcare user. No significant difference in MAE was found among users for HVA (*p* = 0.13), whereas significant differences were noted for some intermetatarsal angles.

**Conclusions:**

The developed smartphone application accurately measured the HVA and various intermetatarsal angles, with performance comparable to that of experienced foot and ankle surgeons. Importantly, sufficient accuracy was achieved even when used by individuals without clinical training. The application may be useful as a practical tool in clinical and research settings.

## 1 Introduction

The hallux valgus angle (HVA) is defined as the angle between the longitudinal axes of the proximal phalanx of the hallux (PH1) and the first metatarsal (MT1) on dorsoplantar foot radiographs, and it reflects the severity of the hallux valgus deformity [1]. The intermetatarsal angle between the first and second metatarsals (M1M2A) is defined as the angle between the axes of MT1 and the second metatarsal (MT2) on dorsoplantar foot radiographs and is also a commonly used parameter for evaluating hallux valgus deformity [1]. These angles are essential for diagnosing hallux valgus, evaluating its severity, and determining the appropriate surgical procedures [2]. Manual measurement methods for these angles were standardized and validated by the Ad Hoc Committee of the American Orthopedic Foot and Ankle Society (AOFAS) in 2002 [1, 3].

To enable highly reproducible measurements of these parameters, we previously developed a neural network model capable of predicting the axes of PH1 and the first to fifth metatarsals on foot radiographs, allowing automatic calculation of HVA, M1M2A, and other intermetatarsal angles [4]. In that study, the measurement accuracy of the neural network was comparable to that of experienced foot and ankle surgeons, even in cases with severe deformity, including crossover toe and joint destruction. However, the system requires Digital Imaging and Communications in Medicine (DICOM) data as input, limiting its practical usability [4, 5].

To overcome this limitation, we developed an Android application that automatically measures HVA, M1M2A, and other intermetatarsal angles—including those between the first and third, fourth, and fifth metatarsals (M1M3A, M1M4A, and M1M5A)—by capturing foot radiographs displayed on a computer screen using a smartphone camera. With this application, the axes of PH1 and the five metatarsals can be detected within approximately 10 s.

The aim of the study was to evaluate the measurement accuracy of this application by comparing its results with manual measurements performed by experienced foot and ankle surgeons.

## 2 Materials and Methods

### 2.1 Application for Automatic Measurements

The developed application predicts the bone axes of PH1 and the first through fifth metatarsals (MT1‐5) using a neural network applied to images captured by the device's camera. The neural network is based on U‐Net architecture with five encoder–decoder blocks and was validated in our previous study [4, 6].

**FIGURE 1:**
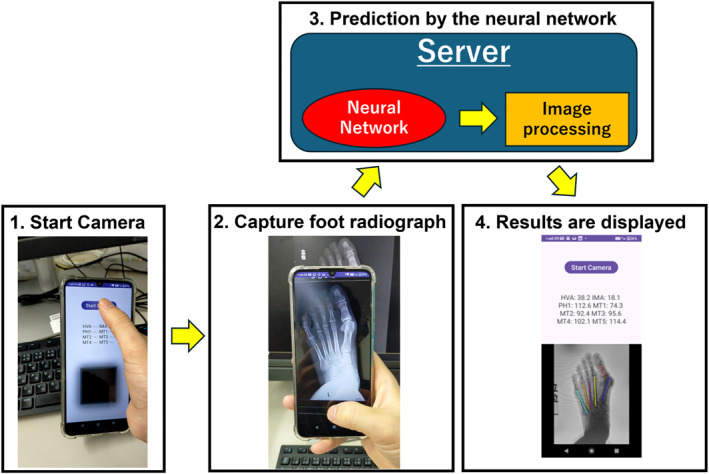
Overview of the application workflow. After the application is launched, camera mode can be started. When a foot radiograph displayed on a PC screen is captured using the smartphone camera, the image is preprocessed into a 512 × 512 matrix and sent to the server. On the server, the neural network predicts the bone axes and estimates angles, including the hallux valgus angle and various intermetatarsal angles.

When the application launches, a “Start Camera” button appears on the home screen. Tapping this button activates the camera mode. When the “Capture” button is tapped, the captured image is sent to a remote server that hosts the neural network and image processing program. After the prediction by the neural network is completed, the result is returned to the device and the captured image superimposing the predicted axis is displayed on the main screen (Figure 1). Simultaneously, the calculated angles, including HVA, M1M2A, M1M3A, M1M4A, and M1M5A, are presented as text (Video S1).

In the current implementation, captured images are not stored on the Android device or the server. The application is not yet publicly available but preparations for its release are in progress.

### 2.2 Measurement by the Application

Three users—User 1 (a board‐certified orthopedic surgeon with more than 10 years of experience), User 2 (an orthopedic resident with half a year of experience), and User 3 (a nonhealthcare professional)—independently performed measurements using the application, each on their own Android devices. The users' characteristics and the specifications of their devices were summarized in Table 1.

**TABLE 1:** Demographics of the users and specifications of their smartphones.

	User 1	User 2	User 3
Age (years)	41	27	36
Sex	Male	Male	Female
Occupation	Attending surgeon	Resident surgeon	Nonhealthcare professional
Smartphone specification			
Device	OUKITEL C51	HUAWEI P30 Lite	OPPO A5 2020
Camera resolution [pixel]	3120 × 4160	2312 × 1080	4000 × 5000
Android version	13	10	10

Users were instructed to capture images as parallel to the screen as possible and to ensure that the entire foot area was included and maximized in the frame. They were allowed to retake images if the predicted axes appeared inappropriate after reviewing the captured image.

Radiographs were displayed on a computer monitor using RadiAnt DICOM Viewer (https://www.radiantviewer.com) with a resolution of 1920 × 1080 pixels and a refresh rate of 60 Hz. The room lighting was dimmed during image capture to mitigate screen glare.

### 2.3 Manual Measurement as a Reference

Manual measurements were performed by three reference foot and ankle surgeons, each with more than 10 years of clinical experience. To establish a reference for evaluating the application's measurement accuracy, the median values of the three raters were used. The raters manually drew the axes of PH1 and MT1‐5 using a DICOM viewer, and the corresponding angles were automatically calculated.

### 2.4 Dataset for the Validation

The data collection for the validation was done by a retrospective cohort. We reviewed the database of first‐visit patients at our foot and ankle outpatient clinic between May 2022 and June 2023 and identified 200 consecutive patients. After excluding patients with rearfoot disease (pathological conditions affecting the talocrural and subtalar joints and surrounding soft tissues, such as ankle osteoarthritis, chronic ankle instability, plantar fasciitis, or Achilles tendinitis)—which are unlikely to have anteroposterior foot radiographs (91 patients), no available foot radiographs (11 patients), foot fractures (4 patients), massive tumoral osteolysis (1 patient), and previous forefoot surgery (1 patient), 92 radiographs from 92 patients were included in the validation cohort. To ensure data independence, one radiograph was selected from each patient even in cases of bilateral involvement. The demographic characteristics and diagnosis of the patients were described in Table 2.

**TABLE 2:** Demographic characteristics of patients included in the validation cohort.

Age (years)	61.7 ± 17.4
Sex (male/female)	20/72
Side (right/left)	52/40
Diagnosis [*n*, (%)]	
Hallux valgus	46 (49%)
Adult‐acquired flatfoot deformity	11 (12%)
Rheumatoid arthritis	10 (11%)
Hallux rigidus	7 (8%)
Plantar plate injury	4 (5%)
Sesamoid disorders	4 (5%)
Tumor	3 (5%)
Curly toe	2 (2%)
Psoriatic arthritis	2 (2%)
Others	3 (4%)
Radiographic parameters	
HVA in manual measurements (degrees)	29.9 ± 14.6
M1M2A in manual measurements (degrees)	14.3 ± 4.3

*Note:* Age is presented as mean ± standard deviation. The category “Others” contains one case each of osteomyelitis, complex regional pain syndrome, and neuropathy of unknown cause.

Abbreviations: HVA, hallux valgus angle; M1M2A, intermetatarsal angle between the first and second metatarsals.

### 2.5 Outcomes and Statistical Analysis

The primary outcome was the mean absolute error (MAE) compared to the manual measurements. MAEs for the three application users were statistically tested against a threshold of 3° using one‐tailed t‐tests, based on the criterion that errors below 3° are considered good or excellent according to a previous study validating the manual measurement [3]. Bland–Altman plots were also generated to visualize the distribution of measurement errors.

The secondary analysis compared MAEs among the three users using analysis of variance (ANOVA). When ANOVA indicated significant differences, multiple comparisons were conducted using the Tukey–Kramer method. Statistical significance was set at *p* < 0.05.

### 2.6 Software Environment

The application was developed using Android Studio Jellyfish. The minimum requirement of Android API level was 24. Statistical analyses were performed using MATLAB R2024a (MathWorks Inc., NA, USA).

## 3 Results

### 3.1 Measurement Errors of Application Users Compared to Manual Measurements

**FIGURE 2:**
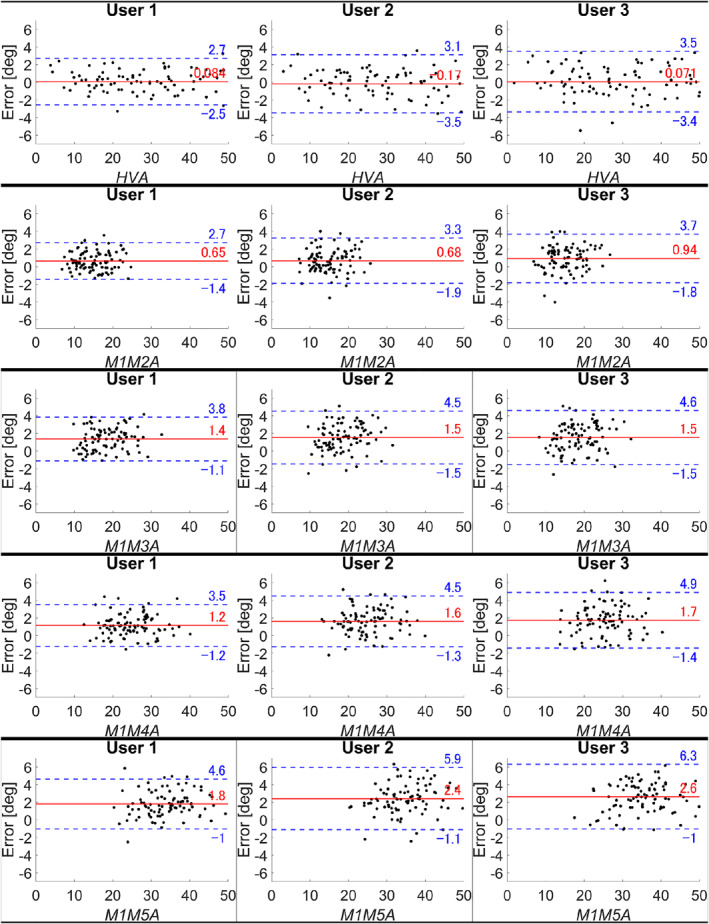
Bland–Altman plots for hallux valgus angle and intermetatarsal angles. The plots show the measurement errors between manual and automatic measurements. The *x*‐axis represents the manual measurement for the parameter. The *y*‐axis represents the difference calculated as follows: manual measurement minus automatic measurement. The red solid line indicates the mean difference, and the blue dashed lines represent the upper and lower limits of agreement defined as the mean ± 1.96 standard deviations. HVA, hallux valgus angle; M1M2A‐M1M5A, intermetatarsal angles between the first and second to fifth metatarsals.

The MAEs for all parameters measured by User 1, User 2, and User 3 were showcased in Table 3. All MAEs except for M1M5A measured by User 3 were significantly smaller than three degrees. The Bland–Altman plots illustrating the measurement errors for all parameters were presented in Figure 2.

**TABLE 3:** Differences between automatic and manual measurements.

	User 1		User 2		User 3	
	MAE (95% CI)	*p*‐value	MAE (95% CI)	*p*‐value	MAE (95% CI)	*p*‐value
HVA	1.1 (1.2)	< 0.01	1.3 (1.5)	< 0.01	1.4 (1.5)	< 0.01
M1M2A	1.0 (1.1)	< 0.01	1.1 (1.3)	< 0.01	1.4 (1.5)	< 0.01
M1M3A	1.5 (1.7)	< 0.01	1.8 (2.0)	< 0.01	1.8 (2.0)	< 0.01
M1M4A	1.3 (1.5)	< 0.01	1.8 (2.0)	< 0.01	2.0 (2.2)	< 0.01
M1M5A	1.9 (2.1)	< 0.01	2.6 (2.8)	< 0.01	2.7 (3.0)	0.07

*Note:* The *p*‐values and upper limits of the 95% confidence intervals are based on one‐sample *t*‐test to test for a one‐tailed MAE of less than 3°.

Abbreviations: CI, confidence interval; HVA, hallux valgus angle; M1M2A‐M1M5A, intermetatarsal angles between the first and second to fifth metatarsals; MAE; mean absolute error.

### 3.2 Comparison of Measurement Errors Among Users With Different Levels of Experience

No significant difference in MAE was observed among the three users for HVA and M1M3A (HVA: *p* = 0.13 and M1M3: *p* = 0.13). In contrast, significant differences were found for M1M2A, M1M4A, and M1M5A (all *p* < 0.01).

**FIGURE 3:**
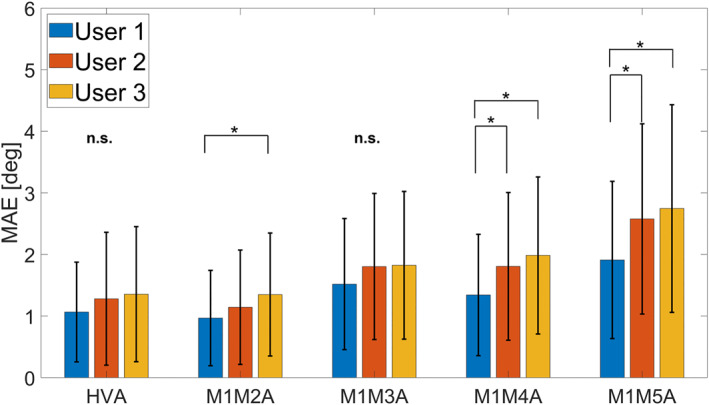
Comparison of mean absolute errors among users. Bar graphs show the mean absolute errors (MAEs) for each parameter across the three users. Multiple comparisons were performed using one‐way analysis of variance followed by the Tukey–Kramer method. Asterisks indicate statistically significant differences at *p* < 0.05. HVA, hallux valgus angle; M1M2A–M1M5A, intermetatarsal angles between the first and second to fifth metatarsals; n.s., not significant.

For M1M2A, the MAE of User 1 (experienced surgeon) was significantly smaller than that of User 3 (nonhealthcare professional). For M1M4A and M1M5A, the MAEs of User 1 were significantly smaller than those of both User 2 (resident) and User 3 (nonhealthcare professional) (Figure 3).

## 4 Discussion

The current study evaluated the reproducibility of measurements obtained using a smartphone camera application against the median of manual measurements performed by three experienced reference foot and ankle surgeons. In addition, interoperator reliability was assessed among three users with different levels of clinical experience. Although interoperator reliability varied for some parameters, the measurements obtained by all application users demonstrated sufficient reproducibility compared with the median of manual measurements.

The current study demonstrated that a neural network was capable of extracting bone axes from foot radiographs displayed on a monitor and photographed using a smartphone camera. Previous studies have explored the application of artificial intelligence (AI) to smartphone‐captured radiographs, primarily in the field of chest imaging. For example, retraining neural networks with smartphone‐captured chest radiographs has been shown to improve detection performance, even when smartphone images are used as input [7]. Similarly, high accuracy has been reported in identifying and classifying cardiac devices from camera‐captured chest radiographs [8]. However, these tasks were relatively simpler compared to performing automatic geometric measurements.

A previous study conceptually similar to ours was conducted using an application called *Hip Scouter* (Zimmer‐Biomet, IN, USA), which allows users to photograph hip radiographs displayed on a screen and utilizes AI to calculate implant alignment angles [9]. Although that study also reported a high correlation between AI and manual measurements, it focused on prosthetic geometry, which offers high‐contrast landmarks. Therefore, a lightweight model, such as You Only Look Once version 5 (YOLOv5), was sufficient for accurate measurements. In contrast, our study involved plain foot radiographs, which lack distinct landmarks and show greater anatomical variability. As a result, we employed a more complex neural network that could not be embedded in a smartphone and performed the computations on a remote server to ensure accurate bone axis extraction.

The current study demonstrated high consistency between the application's measurements and manual measurements by the three reference foot and ankle surgeons across all parameters. The parameter with the lowest accuracy was M1M5A measured by a nonhealthcare professional; however, the MAE remained at 2.7°, with an upper 95% confidence limit of 3.0°, which is within the commonly accepted threshold for good agreement. The MAEs for HVA and M1M2A ranged from 1.1° to 1.4°, indicating excellent agreement with manual measurements. One factor contributing to this high accuracy is the algorithm of the angle estimation itself. Rather than segmenting bone regions or detecting key anatomical landmarks, the neural network was trained to directly predict the orientation of the bone axes as line segments. This approach reduced ambiguity in the annotation process and minimized the influence of overlapping bones or image artifacts, leading to more stable and consistent predictions. Our previous study using DICOM data as input reported MAEs of 1.3° for HVA and 0.8° for M1M2A [4]. These values are markedly lower compared to those reported in other previous studies [10, 11, 12]. Moreover, although most previous validation studies excluded radiographs with severe deformities or joint destruction, both the current and our previous studies included such cases with joint dislocations or rheumatoid arthritis. Another contributing factor is the preprocessing of the input images. Our neural network begins by preprocessing DICOM files into a 512 × 512 grayscale matrix, where pixel values are standardized to a range of 0–1 [4]. This standardization and simplification allowed us to directly repurpose the model for smartphone‐captured images by converting them into the same format.

The current study showed that the measurement accuracy tended to decrease in the order of HVA, M1M2A, M1M3A, M1M4A, and M1M5A. Based on the Bland–Altman plot, the application measurements tended to be smaller than the manual measurements, particularly for M1M3A, M1M4A, and M1M5A. Since this tendency was not observed when using DICOM inputs in our previous study [4], it is reasonable to attribute the discrepancy to the use of camera‐captured images. When the camera was tilted inward or outward relative to the screen, the angles between two lines appeared smaller, with the effect being more pronounced for larger angles. In contrast, the impact was less evident when the line segments forming the angle were shorter as observed in the HVA measurement. If the automatic measurements for M1M3A, M1M4A, and M1M5A were adjusted using a keystone correction algorithm, further improvements in accuracy could be expected.

The current study showed that the MAEs of M1M2A, M1M4A, and M1M5A differed among the application users with varying levels of clinical experience. In a preceding study evaluating the accuracy of the Hip Scouter application, differences between users were not investigated [9]. In our study, User 1, an experienced orthopedic surgeon, had the smallest MAE in all parameters. One possible explanation is that users were allowed to repeat the measurements at their discretion, which enabled the experienced surgeon to identify and exclude inaccurate measurements, including those resulting from suboptimal image acquisition conditions, such as monitor refresh artifacts or glare that interfered with accurate bone axis estimation. Another contributing factor may be the variation in users' ability to position the smartphone camera as parallel to the screen as possible—a technique that significantly affects measurement accuracy.

There were some limitations in the study and the application. First, the performance assessment was conducted at a single institution, and all radiographs were obtained at the same hospital. Although it is unlikely that institution‐specific information was embedded in the 512 × 512 pixel matrices obtained through image capture and preprocessing given the standardized input format, differences in institutional radiographic protocols, such as patient positioning, beam angles, and equipment calibration, could influence image quality and potentially affect model performance. Second, a limitation of the application is that it cannot currently be applied to postoperative radiographs. This is because the definition of the MT1 axis used for postoperative assessment differs from that used for preoperative measurements in the manual method recommended by the AOFAS [1]. Developing a separate model trained on datasets that include postoperative radiographs from various osteotomy procedures will be necessary in future work. Third, there may be institutional or regulatory restrictions in certain healthcare settings because the use of the application involves capturing radiographs displayed on clinical workstation monitors using smartphones. Although images captured by the application are neither stored on the smartphone nor saved on the server to which they are transmitted, future users should be made aware of any such restrictions and comply with institutional policies when using the application.

## 5 Conclusion

We developed an Android application capable of automatically measuring HVA and various intermetatarsal angles. The results closely matched manual measurements performed by the three reference foot and ankle surgeons. Although there were differences among the three application users—one board‐certified orthopedic surgeon, one orthopedic resident, and one nonhealthcare professional— all achieved sufficient measurement accuracy. This camera‐based application does not require integration with PC‐based software, such as DICOM viewers. Its simplicity and reproducibility make it a practical tool for both routine clinical use and research involving foot radiographs.

## Author Contributions

**Ryutaro Takeda:** conceptualization, formal analysis, funding acquisition, software, writing – original draft. **Sanehiro Ando:** data curation (equal), **Toshiko Iidaka:** data curation (equal), resources (equal). **Kenta Makabe:** data curation (equal), resources (equal). **Taro Kasai:** data curation (equal), resources (equal). **Yasunori Omata:** investigation. **Noriko Yoshimura:** data curation (equal), resources (equal). **Sakae Tanaka:** project administration. **Takumi Matsumoto:** methodology, supervision, writing – review and editing.

## Ethics Statement

This study was approved by the Research Ethics Committee, Graduate School of Medicine and Faculty of Medicine, The University of Tokyo (No. 2674‐4).

## Consent

Informed consents were obtained from all patients in the form of opt‐out on the hospital website.

## Conflicts of Interest

The authors declare no conflicts of interest.

## Supporting information


**Video S1:** Measurement process using the Android application. By tapping the capture button to photograph the foot radiograph displayed on a PC monitor, the image is transmitted to the server, where the AI generates the estimated bone axis image and calculates the angles of each bone axis.

## Data Availability

The radiographic data supporting the findings of this study are not publicly available due to privacy and ethical restrictions. However, the data are available from the corresponding author upon reasonable request. The source code of application are not publicly available due to future commercial use.
